# Elevated plasma fibrinogen level shows superior prognostic value than Epstein–Barr virus DNA load for stage IVA/B nasopharyngeal carcinoma patients in the intensity-modulated radiotherapy era

**DOI:** 10.18632/oncotarget.10083

**Published:** 2016-06-15

**Authors:** Mei Lan, Chunyan Chen, Ying Huang, Minjie Mao, Fei Han, Junfang Liao, Meiling Deng, Zhijun Duan, Lie Zheng, Shaoxiong Wu, Taixiang Lu, Yutao Jian

**Affiliations:** ^1^ Department of Radiation Oncology, State Key Laboratory of Oncology in Southern China, Sun Yat-sen University Cancer Center, Collaborative Innovation Center for Cancer Medicine, Guangzhou, China; ^2^ Department of Clinical Laboratory, State Key Laboratory of Oncology in Southern China, Sun Yat-sen University Cancer Center, Collaborative Innovation Center for Cancer Medicine, Guangzhou, China; ^3^ Department of Imaging Diagnosis and Interventional Center, State Key Laboratory of Oncology in Southern China, Sun Yat-sen University Cancer Center, Collaborative Innovation Center for Cancer Medicine, Guangzhou, China; ^4^ Department of Radiation Diagnosis and Interventional Center, Chengdu Military General Hospital, Chengdu, China; ^5^ Institute of Stomatological Research, Sun Yat-sen University, Guangdong Provincial Key Laboratory of Stomatology, Guangzhou, China

**Keywords:** fibrinogen, nasopharyngeal carcinoma, stage IVA/B disease, Epstein–Barr viral DNA, prognosis

## Abstract

**Purpose:**

Effective prognostic factors for patients with stage IVA/B nasopharyngeal carcinoma (NPC) who are susceptible to distant metastases are limited. We aim to investigate the prognostic value of pretreatment plasma fibrinogen (FIB) level and Epstein–Barr virus DNA (EBV-DNA) load in these patients in the era of intensity-modulated radiotherapy (IMRT).

**Results:**

The 5-year DSS, DFS and DMFS rates of the entire cohort were 72.7%, 66.8%, 80.0%, respectively. High FIB level was identified as a negative prognostic factor for survival: the 5-year DSS, DFS and DMFS rates for patients with high FIB (> 4.0 g/L) and normal FIB (≤ 4.0 g/L) were 60.3% vs. 76.0%, 56.0% vs. 69.9%, and 59.4% vs. 85.5%, respectively (all *P* < 0.001). Subgroup analysis demonstrated that DSS, DFS and DMFS decreased as FIB gradually increased, even within the normal range. The risk of distant metastasis in patients with high FIB was over 3-fold than patients with normal FIB. EBV-DNA was not an independent prognostic factor for any survival outcomes in multivariate analysis.

**Conclusion:**

High pretreatment FIB level shows superior prognostic value than EBV-DNA load for stage IVA/B NPC patients in the era of IMRT.

**Materials and Methods:**

A total of 755 patients with newly-diagnosed stage IVA/B NPC treated with definitive IMRT between January 2007 and December 2011 were enrolled. Plasma FIB and EBV-DNA were measured before treatment. Disease-specific survival (DSS), disease-free survival (DFS) and distant metastasis-free survival (DMFS) were calculated using the Kaplan-Meier method; differences were compared using the log-rank test.

## INTRODUCTION

Nasopharyngeal carcinoma (NPC), a malignant tumor type with a distinct geographical and ethnic pattern of incidence, is most common in southern China. Due to its atypical symptoms and concealed location, almost one third of patients are diagnosed with stage IVA/B disease. The prognosis of stage IVA/B disease is much poorer than stage III disease and the 5-year overall survival rate is still around 60% to 70%, even though intensity-modulated radiotherapy (IMRT) has been widely used. [1–3] Therefore, it is important to identify effective prognostic factors for this subgroup of patients.

Coagulation and activation of the fibrinolytic system are frequently observed in patients with cancer, and are associated with higher risks of progression, metastasis and poor outcome. [4–6] A relationship between cancer, hemostasis and inflammation has been widely accepted and over-expression of a number of procoagulant and fibrinolytic factors, especially fibrinogen (FIB), has been reported in malignant tumors. [4, 5] Extensive studies indicate a specific correlation between high plasma FIB level and the progressive and metastatic behaviour of tumour cells. [7, 8] An elevated pretreatment plasma FIB level has been identified as a significant negative prognostic factor for poorer survival in various solid tumour types, including lung, breast, oesophageal cancers and NPC. [6, 9–14] However, for NPC patients, findings from previous study [6] were based on stage I-IV diseases and different radiotherapy techniques. The role of pretreatment FIB level in stage IVA/B NPC patients treated with IMRT remains unknown.

Although pretreatment Epstein–Barr virus DNA (EBV-DNA) load has been regarded as a useful diagnostic biomarker for NPC for many years [15–18]. However, in our clinical experience, unlike many patients who have stage III diseases, patients with stage IVA/B diseases usually have extremely high EBV-DNA loads. Whether elevated EBV-DNA remains to be an effective prognostic biomarker for stage IVA/B patients deserves further investigation.

Therefore, we conducted a retrospective study of a large cohort of patients to investigate the prognostic value of pretreatment plasma FIB level and EBV-DNA loads in stage IVA/B NPC in the era of IMRT.

## RESULTS

### Survival outcomes of patients with different pretreatment plasma FIB levels

**Figure 1:**
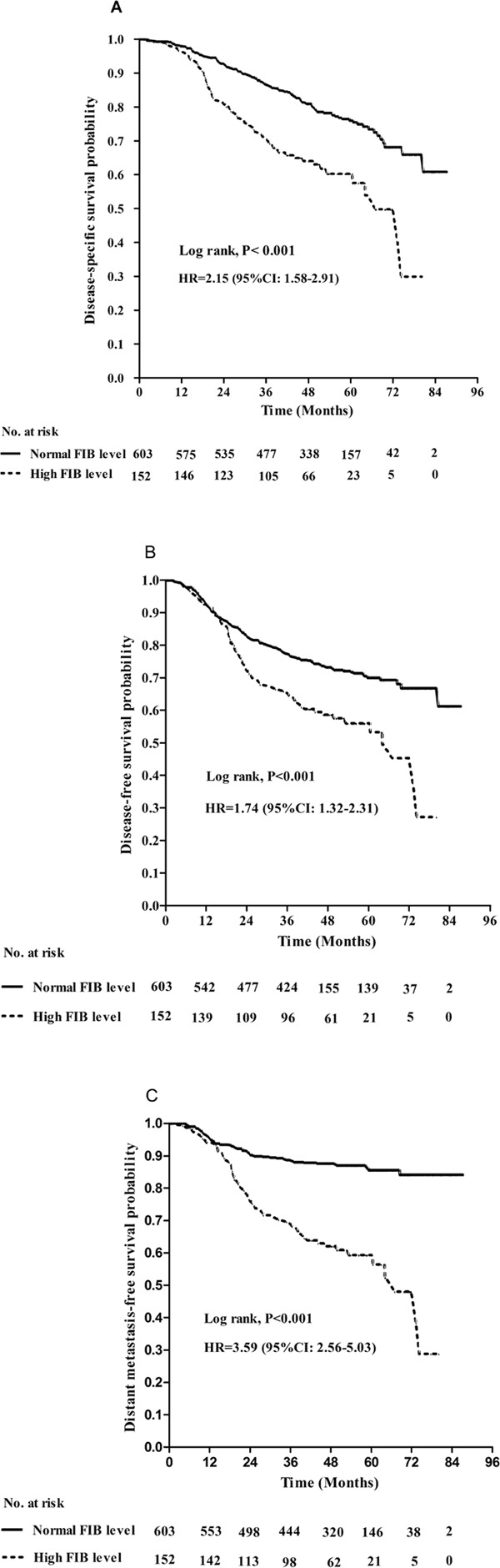
Kaplan–Meier **A.** disease-specific survival, **B.** disease-free survival and **C.** distant metastasis-free survival curves for patients with stage IVA/B NPC stratified by pretreatment plasma FIB (> 4 g/L vs. ≤ 4 g/L) *P*-values were calculated using the unadjusted log-rank test; Hazards ratios (HR) and 95% confidence intervals (CIs) were calculated using the unadjusted Cox proportional hazards model.

Within a median follow-up period of 50 months (range, 3-87 months), 194/755 (25.7%) patients died and 236/755 (31.3%) patients developed disease progression. One fifth of patients (20.1%, 152/755) had high plasma FIB (> 4.0 g/L) levels, of whom 61/152 (40.1%) died and 68/152 (44.7%) suffered treatment failure. The 5-year DSS, DFS and DMFS rates for all patients were 72.7%, 66.8%, 80.0%, respectively. The rates of 5-year DSS, DFS and DMFS for patients with high FIB levels were significantly poorer than those of patients with normal FIB levels (60.3% and 76.0%, 56.0% vs. 69.9%, 59.4% and 85.5%, respectively; all *P* < 0.001; Figure 1).

Patients with stage IVA NPC had a better prognosis than patients with stage IVB NPC, with 5-year DSS, DFS and DMFS rates of 74.7% vs. 69.2% (*P* = 0.042), 69.3% vs. 62.5% (*P* = 0.022), and 82.1% vs. 76.0% (*P* = 0.009), respectively. Survival analysis of the patients with stage IVA NPC showed that high FIB level was associated with significantly poorer DSS, DFS and DMFS than normal FIB level (all *P* < 0.001). High FIB level was also associated with poorer DMFS (*P* < 0.001), but not DSS or DFS, compared to normal FIB in patients with stage IVB NPC (Table 1).

**Table 1:** Survival analysis of patients with stage IVA or IVB nasopharyngeal carcinoma stratified by different pretreatment plasma FIB levels

	Stage IVA (*n* = 466)		*P*-value^*^	Stage IVB (*n* = 289)		*P*-value^*^
	High FIB level (n=98)	Normal FIB level (n=368)		High FIB level (*n*=54)	Normal FIB level (*n*=235)	
**5y-DSS**	58.3%	79.1%	<0.001	63.0%	70.8%	0.096
**5y-DFS**	57.1%	72.6%	<0.001	53.7%	64.7%	0.081
**5y-DMFS**	62.3%	87.5%	<0.001	53.6%	82.0%	<0.001

FIB = fibrinogen; High FIB level = FIB concentration > 4.0 g/L; Normal FIB level = FIB concentration ≤ 4.0 g/L; DSS = disease-specific survival; DFS = disease-free survival; DMFS = distant metastasis-free survival;;

* *P*-values were calculated using the log-rank test.

**Figure 2:**
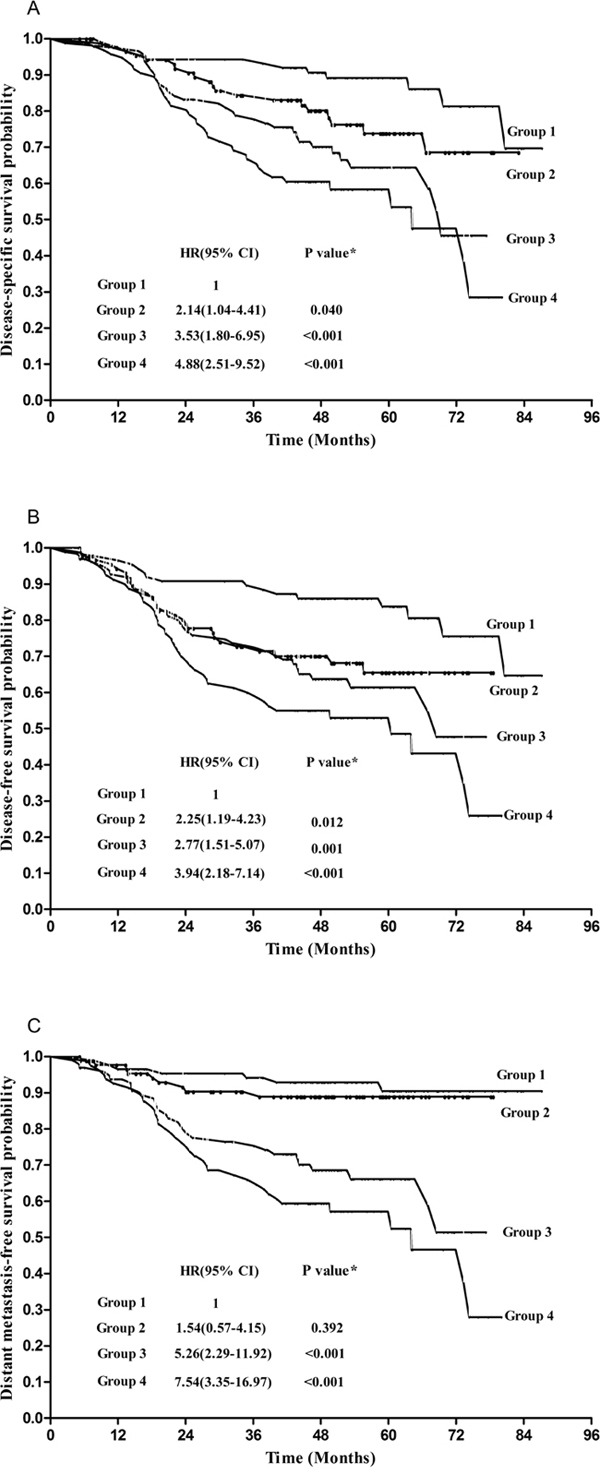
Kaplan-Meier survival curves to compare the disease-specific survival **A.**, disease-free survival **B.**, and distant metastasis-free survival **C.** for patients stratified by the pretreatment plasma FIB quartile values Group 1: < 2.8g/L; Group 2: 2.8-3.6g/L; Group 3: 3.7-4.6g/L; Group 4: >4.6g/L. Hazards ratios (HR), 95% confidence intervals (CIs) and p values were calculated using the unadjusted Cox proportional hazards model.

To further assess the prognostic value of FIB, we divided all patients into four equally-sized subgroups using the upper quartile, median, and lower quartile FIB levels, as follows: < 2.8 g/L, 188 cases (Group 1); 2.8-3.6 g/L, 194 cases (Group 2), 3.7-4.6 g/L, 189 cases (Group 3); > 4.6 g/L, 184 cases (Group 4). Survival curves for the four subgroups are shown in Figure 2. Significant associations were observed between increased FIB and reduced DSS, DFS and DMFS (all *P* < 0.05). Small increases in plasma FIB, even within the normal range, had a significant negative prognostic impact on survival, which indicates that gradual increases in pretreatment plasma FIB are associated with poorer survival outcomes.

### Treatment outcomes for patients with different pretreatment EBV-DNA loads

**Figure 3:**
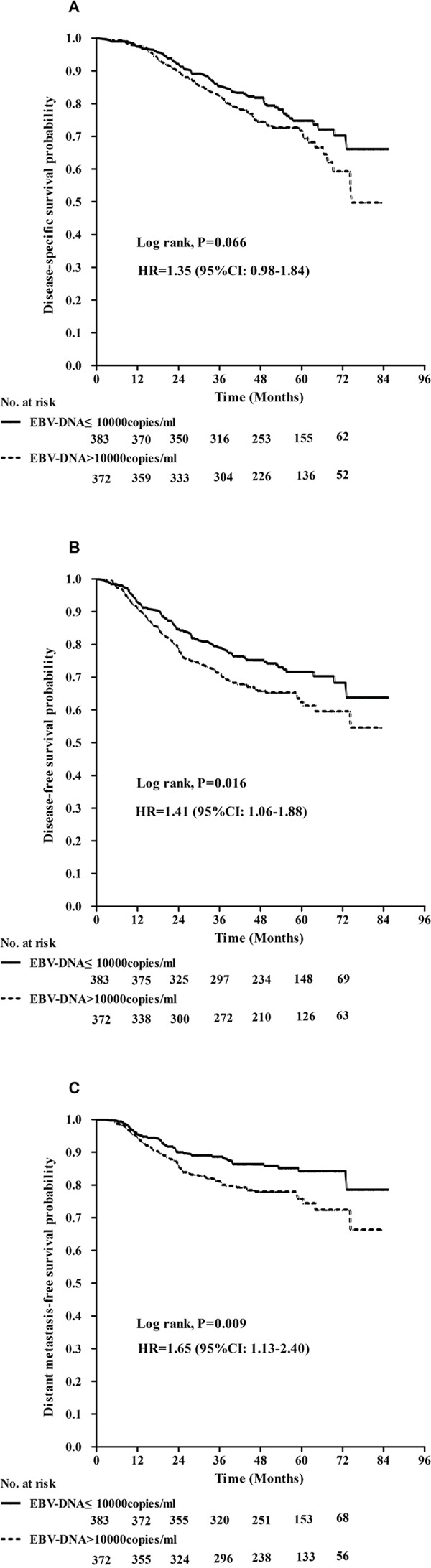
Kaplan–Meier curves show disease-specific survival **A.**, disease-free survival **B.**, and distant metastasis-free survival **C.** between patients with EBV-DNA ≤10000 copies/ml and EBV-DNA >10000 copies/ml P values were calculated using an unadjusted log-rank test.

The median EBV-DNA load was 10000 copies/ml (range, 0-10600000 copies/ml). The 5-year DFS and DMFS rates of patients with an EBV-DNA load > 10000 copies/ml were inferior to those of patients with an EBV-DNA load ≤ 10000 copies/ml (62.0% vs. 71.4%, 75.8% vs. 84.2%, respectively, *P* < 0.02); however, no significant difference in DSS was observed between patients with high and low EBV-DNA loads (*P=0.066*). (Figure 3)

**Figure 4:**
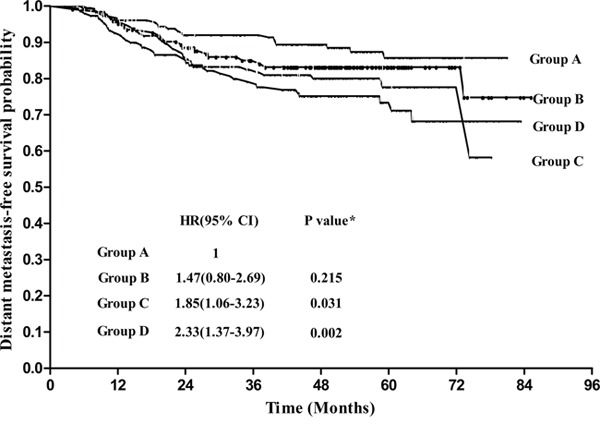
Distant metastasis-free survival for patients grouped according to pretreatment plasma EBV-DNA levels Group A: <1000copies/ml; Group B: 1000-9999 copies/ml; Group C: 10000-50000copies/ml; Group D: >50000copies/ml. Hazards ratios (HR), 95% confidence intervals (CIs) and p values were calculated using the unadjusted Cox proportional hazards model.

To further evaluate the prognostic value of EBV-DNA, we then divided all patients into four equally-sized subgroups according to the upper quartile, median, and lower quartile EBV-DNA loads, as follows: < 1000 copies/ml, 199 cases (Group A); 1000-9999 copies/ml, 188 cases (Group B); 10000-50000 copies/ml, 186 cases (Group C), > 50000, 182 cases (Group D). DMFS slightly reduced as the EBV-DNA load increased, and the survival curves were very close to each other (Figure 4); however, EBV-DNA load was not significantly associated with DSS or DFS (all *P* values >0.05).

### Univariate and multivariate analyses

Univariate analysis of all 755 patients indicated that FIB, EBV-DNA, N category, clinical stage, age (> 45 years) and lactic acid dehydrogenase (LDH) were prognostic factors (Table 2). Multivariate analysis showed that the risk of distant metastasis in patients with high FIB level was over 3-fold than patients with normal FIB level. LDH > 245u/L was an independent negative prognostic factor for DSS and DFS; N stage was an independent prognostic factor for DMFS; and age > 45 years was an independent prognostic factor for DSS. EBV-DNA was not an independent prognostic factor for any survival outcome in multivariate analysis (Table 3).

**Table 2:** Univariate analysis of the associations between clinicopathological variables and clinical endpoints

	5-year DSS (%)	*P*-value^†^	5-year DFS (%)	*P*-value^†^	5-year DMFS (%)	*P*-value^†^
**Age (years)**		0.041		0.193		0.911
≤ 45	75.6		68.0		79.4	
> 45	69.6		65.6		80.6	
**Gender**		0.111		0.168		0.264
Male	71.1		65.4		79.2	
Female	78.1		71.3		82.4	
**T category**		0.441		0.645		0.142
T1	64.2		58.8		69.7	
T2	80.7		72.6		86.8	
T3	72.0		64.4		76.5	
T4	72.1		66.9		80.3	
**N category**		0.121		0.101		0.015
N0	79.6		72.7		86.3	
N1	75.1		70.8		85.0	
N2	72.6		66.4		77.1	
N3	69.2		62.5		76.0	
**Clinical stage**		0.042		0.022		0.009
IVa	74.7		69.3		82.1	
IVb	69.2		62.5		76.0	
**FIB level**		<0.001		<0.001		<0.001
High level	60.3		56.0		59.4	
Normal level	76.0		69.9		85.5	
**EBV-DNA (copies/ml)**		0.066		0.016		0.009
> 10000	71.7		62.0		75.8	
≤ 10000	74.8		71.4		84.2	
**LDH (U/L)**		0.002		0.001		<0.001
> 245	61.8		53.7		66.2	
≤ 245	74.9		68.5		81.8	

DSS = disease-specific survival; DFS = disease-free survival; DMFS = distant metastasis-free survival; FIB = fibrinogen; High FIB level = FIB concentration > 4.0 g/L; Normal FIB level = FIB concentration ≤ 4.0 g/L; EBV-DNA = Epstein-Barr virus DNA; LDH = lactic acid dehydrogenase;

† *P*-values were calculated using the unjusted log-rank test.

**Table 3:** Summary of multivariate analysis of the associations between clinicopathological variables and clinical endpoints

Characteristic	5y-DSS		5y-DFS		5y-DMFS	
	HR (95% CI)^†^	*P*-value^*^	HR (95%CI)^†^	*P*-value^*^	HR (95% CI)^†^	*P*-value^*^
**High FIB level**	1.94 (1.41-2.68)	<0.001	1.51 (1.09-2.10)	0.013	3.17 (2.14-4.69)	<0.001
**LDH > 245 U/L**	1.59 (1.06-2.39)	0.026	1.60 (1.07-2.39)	0.022	1.57 (0.95-2.58)	0.076
**Age > 45 years**	1.37 (1.02-1.83)	0.036				
**N category**					1.53 (1.00-2.35)	0.049
**EBV-DNA>10000copies/ml**			1.26 (0.94-1.70)	0.125	1.26 (0.92-1.65)	0.128

DSS = disease-specific survival; DFS = disease-free survival; DMFS = distant metastasis-free survival; FIB = fibrinogen; High FIB level = FIB concentration > 4.0 g/L; Normal FIB level = FIB concentration ≤ 4.0 g/L; LDH = lactic acid dehydrogenase;

† Hazard ratios;

* *P*-values were calculated using the Cox proportional hazards model.

## DISCUSSION

Our study demonstrates that elevated pretreatment plasma FIB is still associated with poor survival outcomes in stage IVA/B NPC in the era of IMRT; subgroup analysis confirmed DSS, DFS and DMFS decreased as FIB gradually increased, even within the normal range. Patients in our study had relatively high EBV-DNA values, with a median value of 10000 copies/ml (range, 0-10600000 copies/ml). Although EBV-DNA is established as one of the most effective prognostic factors in NPC, [15] it was not confirmed as an independent prognostic factor in this study of patients with stage IVA/B NPC treated with IMRT, indicating the prognostic value of EBV-DNA load should be reconsidered in this specific group of patients.

FIB is an acute phase glycoprotein traditionally associated with the maintenance of hemostasis that has been proven to be a significant prognostic factor in several solid tumor types. [6, 9, 10, 12–14, 19] Tang et al. [6] reported that increased EBV-DNA and fibrinogen levels alone and in combination are associated with reduced survival rates in stage I-IV NPC patients treated with either conventional radiotherapy, three-dimension conformal radiotherapy or IMRT. Our study reconfirmed that the gradual increases in FIB were associated with poorer survival outcomes in stage IVA/B diseases in the era of IMRT, but failed to confirm the value EBV-DNA load in this group of patients.

Independent prognostic factors, such as T category, clinical stage, gender, EBV-DNA load, established in other studies of locoregionally-advanced NPC (which always contain patients with both stage III and IVA/B diseases), [6, 20] did not show prognostic value in this cohort of patients with stage IVA/B diseases. Several factors may explain these differences. Firstly, with the widespread application of IMRT, prognostic factors for NPC may have changed over time. Secondly, patients with stage IVA/B disease are more likely to develop locoregional recurrence and distant metastasis than patients with stage III disease, and the factors that decrease the survival of patients with stage IVA/B are more complicated. Therefore, the prognostic factors identified for locoregionally-advanced NPC (both stage III and IVA/B) in previous studies may not be applicable to the specific group of patients with stage IVA/B diseases. In addition, with the development of conformal radiotherapy technology, such as IMRT, and the use of chemotherapy, the 5-year overall survival rate for stage III NPC is reported to have reached almost 90%, but remains around 60%-70% for stage IVA/B disease. [3, 21, 22] To some extent, these survival differences may reflect inherent biological variations that affect the value of such prognostic factors in patients with stage III and stage IVA/B NPC.

The mechanisms by which FIB affects the survival of patients with cancer have been intensively researched; the evidence indicates that FIB plays a key role in both inflammation and cancer progression. [23] However, the precise molecular mechanisms underlying the relationship between high pretreatment plasma FIB and poorer survival in patients with solid tumors have not been fully clarified.

Consistent with results of previous studies [24, 25], elevated LDH was confirmed as a prognostic factor for poorer survival in this cohort of patients with stage IVA/B NPC, further indicating that high LDH may be associated with a larger tumor burden, tumor extension and higher risk of metastasis.

One major limitation of this study is that only pretreatment plasma FIB was evaluated; we did not evaluate the changes in FIB during and after treatment. As most patients underwent several cycles of chemotherapy, all patients received peripherally-inserted central catheters and/or subclavian vein catheters, which could induce coagulation and activation of fibrinolysis. Secondly, this was a retrospective study of patients treated at a single institute; therefore, these results need to be validated in data sets from other institutions and in prospective studies. Furthermore, the median follow-up period was relatively short (50 months); therefore, long-term validation is required.

In conclusion, high pretreatment FIB level is associated with poorer DSS, DFS and DMFS, and presents to be a better prognostic factor than EBV-DNA for patients with stage IVA/B NPC in the IMRT era.

## MATERIALS AND METHODS

### Patients and work-up

This retrospective study was approved by the institutional review board. In total, 755 patients with newly-diagnosed stage IVA/B NPC who were treated with definitive IMRT, between January 2007 and December 2011, were analyzed in this study. Patients were restaged according to the American Joint Committee on Cancer/International Union Against Cancer (AJCC/UICC) 2010 staging system. Patients with concomitant disease suspected of influencing plasma FIB, such as severe hypertension, liver disease or blood coagulation disorders, were excluded.

All patients underwent a pretreatment evaluation which consisted of a complete physical examination, hematologic and biochemistry profiles, magnetic resonance imaging (MRI) of the nasopharynx and neck, chest radiography or computed tomography (CT), abdominal ultrasonography, and emission CT or whole-body positron emission tomography/CT. Plasma EBV-DNA and FIB were measured for all patients before treatment.

A higher number of patients had stage IVA (466/755; 61.7%) than stage IVB disease (289/755; 38.3%). The male (*n* = 578)/female (*n* = 177) ratio was 3.3:1 and the median age was 45 years (range, 11–78 years). Pathologically, only 3/755 (0.4%) patients had World Health Organization (WHO) type I disease; the remainder had type II or type III disease (Table 4).

**Table 4:** Characteristics of the 755 patients with stage IVA/B NPC

Characteristic	Number of patients (%)
**Age (years)**	
≤ 45	371 (49.1)
> 45	384 (50.9)
**Gender**	
Male	578(76.6)
Female	177 (23.4)
**WHO type**	
III + II	752 (99.6)
I	3 (0.4)
**T category**	
T1	17 (2.3)
T2	59 (7.8)
T3	147 (19.5)
T4	532 (70.4)
**N category**	
N0	53 (7.0)
N1	232 (30.7)
N2	181 (24.0)
N3	289 (38.3)
**Clinical stage**	
IVA	466 (61.7)
IVB	289(38.3)
**FIB level**	
High FIB level	152 (20.1)
Normal FIB level	603(79.9)

Abbreviations: WHO = World Health Organization; FIB = fibrinogen.

### Measurement of fibrinogen and EBV-DNA

Plasma FIB and EBV-DNA are routinely assessed before treatment in our hospital. A 3 mL blood sample was collected and coagulation function was automatically assessed within 3 h of collection using an Analyzer Sysmex CA7000 (Sysmex Corporation, Kobe, Japan) and reagents in accordance with the SIEMENS AG guidelines (Munich, Germany) (Clauss, 1957; Cook and Ubben, 1990) [6]. According to the instructions, a plasma FIB concentration ≤ 4.0 g/L was considered normal, and > 4.0 g/L was defined as hyperfibrinogenemia or high. Using this cut-off value, the patients were classified into the normal FIB and high FIB groups.

Plasma EBV-DNA load was routinely measured by real-time quantitative PCR (RT-qPCR) before treatment using a method that has previously been described in detail. [15, 18]

### Treatment

All patients received definitive IMRT that covered the nasopharynx and retropharyngeal lymph nodes within the primary target volumes. Whole-neck irradiation was performed in all cases. The details of the radiotherapy technique used at our cancer center have previously been reported. [3, 20] Most patients (723/755; 95.8%) received platinum-based chemotherapy: neoadjuvant (546/755; 72.3%), concurrent (548/755; 72.5%) and adjuvant chemotherapy (33/755; 4.4%).

### Follow-up and statistical analysis

Follow-up was measured from the day of diagnosis to the date of the event or last follow-up visit. All patients were followed up by phone calls for the first 3 months, every 3 months for the next 3 years, every 6 months for the next 2 years, and then annually. Disease-specific survival (DSS), disease-free survival (DFS) and distant metastasis-free survival (DMFS) were calculated using the Kaplan-Meier method, and differences were compared using the log-rank test. DSS was defined as the time interval from diagnosis to death caused by cancer or censorship at last follow-up; DFS, to first observation of progression, death or censorship at last follow-up; and DMFS, to first observation of distant lesions or censorship at last follow-up.

For subgroup analysis, the patients were divided into four groups based on the FIB quartile values. Univariate analysis was conducted using the log-rank test. Variables with *P*-values < 0.05 in univariate analysis were included in the final multivariate survival analysis, which was conducted using the Cox proportional hazards model. Statistical Package for the Social Sciences (SPSS, Chicago, IL, USA), version 20.0 was used for all analyses. Two-tailed *P*-values < 0.05 were considered statistically significant.
